# Negative School Gossip and Youth Adolescents’ Mobile Phone Addiction: Mediating Roles of Anxiety and Experiential Avoidance

**DOI:** 10.3390/ijerph20021444

**Published:** 2023-01-13

**Authors:** Jie Xiong, Can He, Hua Wei

**Affiliations:** 1 Center for Mental Health, Wuhan University of Technology, Wuhan 430070, China; 2 College of Education Science, Hubei Normal University, Huangshi 435000, China; 3 Normal College, Qingdao University, Qingdao 266071, China

**Keywords:** negative school gossip, anxiety, experience avoidance, mobile phone addiction, youth adolescent

## Abstract

Being the target of negative school gossip, a form of relational aggression, has been shown to be associated with psychological and behavioral problems in youth adolescents. Based on the experience avoidance model, this study tested the association between negative school gossip and youth adolescents’ mobile phone addiction, and the serial mediation roles of anxiety and experience avoidance in this relationship. Junior high school students (N = 837; ages 12–15; 50% girls) completed the Negative School Gossip Scale, Anxiety Scale, Acceptance and Action Questionnaire (AAQ-II), and Mobile Phone Addiction Scale in their classrooms. The results of regression-based analyses showed that after controlling for age and gender, (1) negative school gossip was significantly associated with mobile phone addiction; (2) anxiety and experience avoidance each significantly mediated this association; (3) anxiety and experience avoidance serially mediated this association. The results support the experience avoidance model and highlight emotional factors as an internal mechanism by which negative school gossip is associated with youth adolescents’ mobile phone addiction. The results also have implications for preventing and reducing youth adolescents’ mobile phone addiction.

## 1. Introduction

Mobile phones have become an indispensable part of people’s daily lives, study, and work. The official investigation report of 2020 [1] shows that China’s urban minors’ Internet penetration rate was 95.0%, and rural minors’ Internet penetration rate was 94.7%. Among the underage netizens, 92.2% use mobile phones to surf the Internet. The Internet devices with the highest proportion of use by underage netizens are mobile phones. While mobile phones bring great convenience to people’s lives, excessive use of mobile phones can also cause physical and psychological problems, including mobile phone addiction [2]. Mobile phone addiction, also known as mobile phone dependence [3] or problematic mobile phone use [4], is an addictive behavior in which individuals use their mobile phones excessively and compulsively, thereby negatively affecting their psychological, behavioral, and social functioning [5]. 

Studies have shown that mobile phone addiction can have a significant negative impact on individual’s physical and mental health, and mobile phone addiction was also negatively associated with sleep quality, academic performance, and interpersonal relationships [3,6,7]. Studies in different countries have shown that more than 30 percent of teenagers are addicted to mobile phones [8]. Studies have also shown that adolescents are a vulnerable group for mobile phone addiction [9]. Youth adolescents are in a critical period of life development, and it is especially their poor ability to cope with stress and mobile phone addiction that has a significant and far-reaching impact on them [10]. 

Problems in peer relations in the school environment such as peer abuse [8] and deviant peer communication [11] have been shown to be associated with a higher risk of youth adolescents’ mobile phone addiction. However, researchers have seldom examined another important factor in the school environment, namely negative school gossip, in relation to this type of addiction. Negative school gossip is a form of relational aggression in which teachers or classmates spread negative or false information about a student. Being the target of negative school gossip can damage the student’s reputation [12]. It has been shown to associate with greater problems in peer relations, such as alienation, exclusion, and rejection [13]. Negative school gossip has even been shown to increase suicidal thoughts [13]. As a typical mental health problem in the digital age, mobile phone addiction may also be affected by negative school gossip.

Previous research on negative gossip has mainly focused on adults. Research in the field of organizational behavior has shown that negative gossip in the workplace can have a wide negative impact on the target’s individual mental health [14]. Gossip accounts for almost 65% of people’s conversation time and is common in various work settings [15,16]. Because negative gossip tends to be more hidden and intriguing [17], people are often more enthusiastic about spreading negative than positive gossip [12]. This may be entertaining for those who gossip, but it can cause psychological problems such as anxiety and depression for those who are gossiped about [14]. 

Youth adolescents who are gossiped about and rejected by peers may have difficulty meeting their relationship needs, however the hierarchy theory of needs [18] emphasizes relationship needs are as basic needs of human beings. When relationship needs are not met, people find alternative ways to meet these needs [19]. In the current study, we focus on the possibility that behaviors associated with mobile phone addiction provide such an alternative. For example, Chen et al. [11] has determined that when individuals encounter peer rejection, their risk of mobile phone addiction increases [20,21]. Negative school gossip may predict mobile phone addiction for the same reason.

The current study was designed to test the association between youth adolescents’ experience of negative school gossip and mobile phone addiction. In addition, we used the experience avoidance model [22] as a conceptual framework for testing several processes that might mediate this association. 

### 1.1. Anxiety as a Mediator

The test of anxiety as a mediator assumes, first, that negative school gossip is associated with youth adolescents’ anxiety. Although there is no direct evidence of this association, two studies provide indirect evidence. In research on adults, being the target of negative gossip in the workplace was associated with high anxiety and low self-esteem [14]. In addition, Taylor et al. [23] determined that relational peer victimization (including the type of negative school gossip) increased the risk of anxiety for youth adolescents.

The test of mediation also assumes that anxiety is associated with mobile phone addiction. Melumad and Pham [24] determined that the portability and social functions of mobile phones may alleviate individuals’ anxiety. Chu et al. [25] has also showed that highly anxious individuals tend to use mobile phones to escape from problems or to seek self-compensation, and are in turn more likely to fall victim to mobile phone addiction. Previous studies have determined that anxiety is one of the strongest personal predictors of mobile phone addiction [8,26]. Thus, negative school gossip may increase the likelihood of youth adolescents’ mobile phone addiction by increasing their anxiety.

### 1.2. Experience Avoidance as a Mediator

According to the experience avoidance model, many negative behaviors are caused by the avoidance of internal experiences such as emotions, thoughts, and awareness of physical states [27]. People will display experience avoidance behavior even if doing so may be harmful in the long run. For example, drug and alcohol abuse, pathological hoarding, and self-harm are often seen as forms of experience avoidance [27,28]. In the digital age, nearly 65.0% of minors own mobile phones [1]. Through mobile phones, youth adolescents can easily participate in many entertaining and stimulating activities (such as online games and short videos), which can effectively attract their attention and help them avoid negative emotional experiences through distraction [10]. On the other hand, negative school gossip can damage the social image and reputation of the target object, which may finally led to their low self-evaluation and unpleasant experience [29]. However, due to the inadequacy of emotion regulation strategies, youth adolescents will quickly relieve the unpleasant experience by avoidance. Therefore, negative school gossip may increase the possibility of mobile phone addiction by increasing experience avoidance. 

### 1.3. The Serial Mediation Effect of Anxiety and Experience Avoidance

Anxiety and experience avoidance may also be serial mediators of the association between negative school gossip and mobile phone addiction. Evidence of the first and last paths of this serial mediation process was reviewed in earlier sections: negative school gossip predicts both anxiety and experience avoidance, and anxiety and experience avoidance both predict mobile phone addiction. The remaining question is whether these two mediators are associated with each other. A review study showed a significant positive correlation between anxiety and experience avoidance [30]. In addition, He et al. [31] argued that negative feelings resulting from anxiety can exacerbate experience avoidance in adolescents. Based on this evidence, we would expect that youth adolescents who feel anxious are more likely to engage in experience avoidance. Thus, considering the literature, it can be inferred that anxiety and experience avoidance are serial mediators in the association between negative school gossip and mobile phone addiction.

### 1.4. Present Study

This study tested the association between youth adolescents’ experience of negative school gossip and mobile phone addiction and tested a model in which anxiety and experience avoidance are serial mediators of this association. We propose three hypotheses:

**H1.**  *Negative school gossip is positively associated with youth adolescents’ mobile phone addiction.*

**H2.**  *Anxiety and experience avoidance act as individual mediators in the association between negative school gossip and mobile phone addiction.*

**H3.**  *Anxiety and experience avoidance act as sequential mediators in the association between negative school gossip and mobile phone addiction.*

## 2. Materials and Methods

### 2.1. Participants and Procedure 

This research was approved by the Research Ethics Committee of the corresponding author’s institution. We contacted the school administrators and introduced our study carefully. The oral informed consent was obtained from the teachers and parents of the participants. We obtained the informed consent of participates in the beginning of the investigation; the instructions of the questionnaire expound that the information obtained is used only for this study and the questionnaire is anonymous. We assured that all the information they fill in would be kept strictly confidential. The participants completed the questionnaire in approximately 10–15 min. In addition, the participants were informed that their participation is completely voluntary, and they can withdraw from the research if they experience any discomfort in the process. The participants were 7th to 9th grade students in three public ordinary junior high schools in Central China and they all lived in city. Five classes per grade were selected through convenience sampling. The students were asked to complete anonymous, paper-and-pencil questionnaires, which were administered in the classroom by a well-trained researcher (response rete: 97.89%). A total of 855 sets of questionnaires were distributed, and 837 were turned in to the researcher. The average age of all participants was 13.54 years old (SD = 0.82). Among them, there were 418 boys and 419 girls.

### 2.2. Measures 

#### 2.2.1. Negative School Gossip Scale

The Negative School Gossip Scale developed by Wang [13] et al. was used to assess the level of negative school gossip in junior high school students perceived by a teacher or classmate. The scale consists of two items, namely “Someone (such as a teacher or classmate) told others something negative about me at school” and “Someone (such as a teacher or classmate) spread rumors about me in the school.” Each item is rated using a five-point scale (1 = “never” to 5 = “always”). The higher the average score, the more frequently the respondent perceived negative school gossip. This measure was shown in earlier research to have good internal consistency and construct validity in a Chinese youth adolescent sample [29]. In this study, the scale’s Cronbach’s α was 0.76.

#### 2.2.2. Anxiety Scale

The Depression–Anxiety–Stress Scale [32] is a widely used measure which contains three subscales to assess symptoms of depression, anxiety and stress. We assessed students’ anxiety using the seven-item anxiety subscale from the Chinese version of the measure [33]. Representative items are “I feel breathless” and “I feel scared for no reason.” Each item is rated using a four-point scale (0 = “totally disagree” to 3 = “totally agree”). The higher the average score, the more serious the individual’s anxiety is. This measure showed good reliability and validity in a Chinese youth adolescent sample [31]. In this study, the anxiety subscale’s Cronbach’s α was 0.76. 

#### 2.2.3. Acceptance and Action Questionnaire-II

The AAQ-II [34] is a widely used measure of the degree of individual experience avoidance. The seven-item scale includes items such as “My painful experiences and memories make it difficult for me to live a life that I would value” and “My painful memories prevent me from having a fulfilling life.” Each item is rated using a seven-point scale (1= “never” to 7= “always”). The higher the average score, the higher the individual’s experience avoidance. The AAQ-II has been shown to have good reliability and validity [35]. A Chinese version of the scale [36] was used in the current study. The scale’s Cronbach’s α was 0.92 in our sample.

#### 2.2.4. Mobile Phone Addiction Scale

The Mobile Phone Addiction Scale revised by Hong [37] et al. was used in current study. It contains 11 items including “When I’m not playing with my phone, I still think about it in my head” and “I tried to reduce the amount of time I spent on my phone, but I failed.” The students rated the items using a six-point Likert scale (1 = totally disagree to 6 = totally agree). The higher the average score, the more serious the individual’s mobile phone addiction is. It was shown in earlier research to have good internal consistency and construct validity [38]. In this study, the scale’s Cronbach’s α was 0.83.

### 2.3. Statistical Analysis

We first computed Pearson correlations among negative school gossip, anxiety, experience avoidance and mobile phone addiction. Then, we tested the mediation model using the SPSS macro-PROCESS (Model 6) which is used to test complex models including serial mediation. We used the bootstrap method with 5000 bootstrap samples drawn from the dataset to calculate indirect and direct effects and check whether the effects were significant. The 95% confidence interval does not include 0, indicating a significant mediating effect [39].

## 3. Results

### 3.1. Preliminary Analyses

Descriptive statistics (*M* and *SD*) for all variables are reported in Table 1. The correlation matrix, also in Table 1, indicated that the four variables of negative school gossip, anxiety, experience avoidance and mobile phone addiction were significantly and positively associated with each other (see Table 1).

### 3.2. Mediation Analysis

Model 6 in the SPSS macro-PROCESS [39] was used to test the serial mediation effect of anxiety and experience avoidance in the association between negative school gossip and mobile phone addiction. Gender and age were included as control variables. Bootstrap samples with 5000 iterations were used to calculate 95% confidence intervals. 

Table 2 shows that negative school gossip was significantly associated with anxiety (*β* = 0.37, *p* < 0.01) and with experience avoidance (*β* = 0.17, *p* < 0.01). Anxiety was significantly associated with experience avoidance (*β = 0*.63, *p* < 0.001) and with mobile phone addiction (*β* = 0.19, *p* < 0.01). Experience avoidance (*β* = 0.10, *p* < 0.05) and negative school gossip (*β* = 0.11, *p* < 0.01) were significantly associated with mobile phone addiction. This suggests that negative school gossip is a significant risk factor for mobile phone addiction. Hypothesis 1 of this study was supported.

The confidence interval of the mediation effect does not contain 0. As a result, anxiety and experience avoidance have a significant serial mediation effect between negative school gossip and mobile phone addiction. This suggests that anxiety and experiential avoidance not only act as mediating variables alone in the relationship between negative school gossip and mobile phone addiction, but also create a serial mediating effect. Hypotheses 2 and 3 of this study were supported.

**Figure 1:**
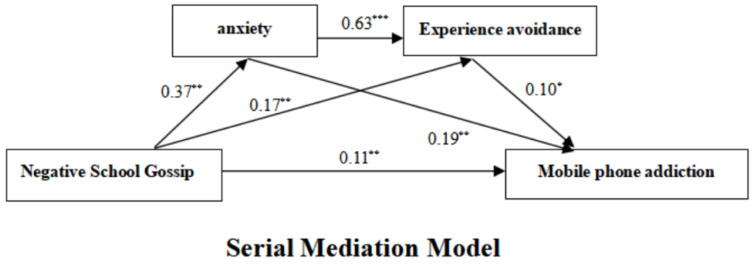
Serial Mediation Model. Note. * *p* < 0.05, ** *p* < 0.01, *** *p* < 0.001.

In addition, as it can be seen from Table 3 and Figure 1, anxiety and experience avoidance mediated the association between negative school gossip and mobile phone addiction. The serial mediation effect (0.11) accounted for 50% of the total effect of negative school gossip on mobile phone addiction (0.22). Specifically, the serial mediation effect consists of indirect effects produced by three paths. Indirect Effect 1: negative school gossip → anxiety → mobile phone addiction. Indirect Effect 2: negative school gossip → experience avoidance → mobile phone addiction. Indirect Effect 3: negative school gossip → anxiety → experience avoidance → mobile phone addiction. The mediation effects for the three models were 0.07, 0.02 and 0.02, respectively. The data in Table 3 show that the three mediation effects accounted for 31.82%, 9.9% and 9.9% of the total effect, respectively. The 95% Confidence Intervals did not contain zero, indicating that all three indirect effects reached a significant level.

## 4. Discussion

Although some factors in the school environment have been shown to be associated with youth adolescent mobile phone addiction, very few studies to date have examined being the target of negative school gossip as a risk factor for this problem behavior. This study explored the effects of negative school gossip on youth adolescent mobile phone addiction, and based on the experience avoidance model, examined anxiety and experiential avoidance as serial mediators of this relationship. For studies on factors influencing mobile phone addiction, previous researchers have mainly explored the role of individual factors such as self-esteem, negative emotions, and self-control [37,40,41], with relatively few examination of the environment such as family factors, school factors, and peer factors [42]. This study contributes to the research field of the influence of school environment on mobile phone addiction.

### 4.1. Negative School Gossip and Youth Adolescents’ Mobile Phone Addiction 

The results showed that being the target of negative school gossip was associated with youth adolescents’ mobile phone addiction, consistent with evidence that peer aggression and deviant peer communication were risk factors for mobile phone addiction [8,11,43]. 

The results suggest that the development of mobile phone addiction can be explained in part by emotional factors in response to negative school gossip as a form of relational aggression. These findings provide support for the experience avoidance model [10], and attention to negative school gossip provides information about the school environment at the micro level. The results can also provide direction for preventing and intervening in mobile phone addiction. 

Schools are the main places for youth adolescents to spend a lot of time in every day, and the relatively closed environment of middle schools is more likely to increase the impact of negative school gossip [13]. Previous studies have determined that negative school gossip is an important external pressure source that causes negative outcomes among junior high school students, such as low self-esteem and mental health problems [29]. As a way of compensation, they will seek nonadaptive cognition in the Internet world continuously and frequently to obtain self-worth [44], while persistent and frequent Internet behavior is likely to lead to mobile phone addiction. Therefore, negative school gossip is one of the risk factors of adolescents’ mobile phone addiction. This study is the first to verify the direct relationship between negative school gossip and mobile phone addiction, enriching the risk factors of mobile phone addiction.

### 4.2. The Mediating Effect and Serial Mediating Effect of Anxiety and Experience Avoidance 

Anxiety and experience avoidance, considered separately, were both significant mediators of the relationship between negative school gossip and mobile phone addiction. In addition, anxiety and experience avoidance were serial mediators, in line with the experience avoidance model [22]. 

First, negative school gossip appeared to increase the risk of mobile phone addiction by triggering anxiety. This is consistent with the results of a previous study showing that school aggression and interpersonal rejection increased the risk of mobile phone addiction through anxiety [20]. Adolescents with negative school gossip will be more worried about others’ evaluation of themselves and more sensitive to interpersonal interaction information, which will generate more anxiety. The overuse of mobile phones to alleviate anxiety will eventually lead to mobile phone addiction. The results of this study further verified the mediating role of anxiety in the formation mechanism of mobile phone addiction [45].

Second, negative school gossip appeared to increase the risk of mobile phone addiction through experience avoidance, consistent with previous studies showing that experience avoidance increases the risk of mobile phone addiction [10]. The various functions of mobile phones enable individuals to temporarily be distracted from the distress caused by negative school gossip and invest in a virtual world with greater autonomy. Therefore, individuals avoid the negative experience of being attacked through mobile phone addiction.

Finally, the results suggest that negative school gossip can increase the risk of mobile phone addiction through the serial mediation of anxiety and experience avoidance, which is in line with the experience avoidance model. The experience avoidance model can well explain the formation mechanism of a variety of adverse behaviors. Previous studies have determined that external stimuli can affect adaptation to negative behaviors (e.g., drug and alcohol abuse, Internet addiction, and self-harm) through the intermediary effects of negative emotions (anxiety, depression) and experience avoidance [27,28,31,46]. Wei et al. [10] also explained the effects of harsh parenting on youth adolescent mobile phone addiction using the experience avoidance model. This is the first study to introduce negative school gossip into the experience avoidance model of mobile phone addiction, and the results further extend the applicability of the theory.

Youth’s mobile phone addiction will be influenced by external environmental factors and internal individual factors [42,47]. There are two main directions for existing interventions on mobile phone addiction. One is based on the change in individual internal factors, such as the neurocognitive processes and coping style [48,49]. The other is intervention based on the change in external environmental factors, such as family and parental control [50] and the early intervention program [47] that need to create a good atmosphere through systematic improvement and coordinated intervention. It will be more effective for us to intervene from an integrated perspective in the future; Du et al. [51] suggested that multimodal school-based group CBT was also a good attempt.

## 5. Conclusions

To summarize, the current study took a critical step in establishing a serial mediation model which provides an elaborate understanding of the relationship of negative school gossip to youth adolescents’ mobile phone addiction. Specifically, we determined that negative school gossip is a risk factor for youth adolescents’ mobile phone addiction. In addition to that, anxiety and experiential avoidance play a significant mediating role between negative school gossip and mobile phone addiction, and the mediating effect includes three paths: the first is the separate mediating effect of anxiety; the second is the separate mediating effect of experience avoidance; and the third is the chain mediation effect of anxiety and experience avoidance.

## 6. Limitations and Implications

This study should be considered in the context of several limitations. First, this study adopted a cross-sectional design, which does not provide information about causality or dynamic changes in the mechanisms of effects. Future studies could adopt longitudinal or experimental studies to provide stronger evidence of the link between negative school gossip and mobile phone addiction, and to further test mechanisms of this link. In addition, the present study only explored the mediating effect of anxiety and experience avoidance between negative school gossip and mobile phone addiction. Maybe there are other similar vulnerability variables; therefore, the possible variables between the two could be studied in the future. Finally, we used a convenience sample, which may restrict the generalizability of our results. Future studies may explore the proposed model among diverse populations. 

The present study has significant practical implications. At present, many middle schools in China focus on improving students’ academic performance and the school’s physical environment, but there is a lack attention to creating a positive emotional and psychological environment. When youth adolescents show a high degree of problematic mobile phone use, their academic performance is bound to be affected [52,53]. Therefore, strengthening the construction of the school’s emotional and psychological environment is crucial to students’ academic achievements and psychological health. Schools can offer lessons and courses to inform youth adolescents about the potential negative consequences of seemingly casual school gossip. 

At the same time, youth adolescents suffering from negative school gossip may benefit from school mental health services. Clinicians could teach emotion regulation strategies to reduce anxiety in response to the gossip. Another possibility is to teach cognitive re-evaluation rather than expression suppression [54]. Acceptance Commitment Therapy (ACT) may also be useful, which emphasizes the acceptance of internal experience as the basic for problem-solving [55,56]. Reducing experience avoidance is a cornerstone of this therapy approach. In this model, acceptance does not mean enduring or yielding, but rather getting out of trouble through value commitment and goal action based on recognizing one’s inner experience. The ACT effectively improved Chinese adolescents’ psychological flexibility, which contains the process of acceptance [57]. Therefore, schools can adopt acceptance therapy to help youth adolescents distressed by negative school gossip to reduce experience avoidance with the goal of reducing problem behaviors such as mobile phone addiction.

## Figures and Tables

**Table 1:** Statistics and correlation analysis between variables.

Variable	*M*	*SD*	1	2	3	4
1. Negative school gossip (NSG)	1.56	0.73	—			
2. Anxiety	0.70	0.66	0.36 ***	—		
3. Experience avoidance (EA)	2.15	1.19	0.40 ***	0.69 ***	—	
4. Mobile phone addiction (MPA)	3.00	1.01	0.21 ***	0.31 ***	0.28 ***	—

Note: *M* is short for the mean, *SD* is short for the standard deviation; *N* = 837, *** *p* < 0.001. NSG = Negative school gossip, EA = Experience avoidance, MPA = Mobile phone addiction.

**Table 2:** Regression analysis of variable relationships in the mediation model.

Regression Equation			Exponential Fitting			Significant Test of Regression Coefficient
Outcome	Predictor	R	R^2^	F	β	t
Anxiety	Gender	0.38	0.14	45.63 **	0.18	2.65 **
	Age				0.09	2.81 **
	NSG				0.37	11.41 **
EA	Gender	0.71	0.50	216.42 **	0.13	2.54 **
	Age				−0.02	−0.71
	NSG				0.17	6.65 **
	Anxiety				0.63	23.99 ***
MPA	Gender	0.34	0.12	23.34 **	0.06	0.90
	Age				0.09	2.88 **
	NSG				0.11	2.96 **
	Anxiety				0.19	4.26 **
	EA				0.10	2.09 *

Note. *N* = 837, * *p* < 0.05, ** *p* < 0.01, *** *p* < 0.001.

**Table 3:** 95% Confidence Intervals of the Mediation Effects.

Path	Standard Estimate	Boot 95% CI		Mediating Effect
NSG → Anxiety → MPA	0.07	0.04	0.11	31.82%
NSG → EA → MPA	0.02	0.002	0.03	9.9%
NSG → Anxiety → EA → MPA	0.02	0.002	0.04	9.9%

## Data Availability

To protect the participants’ privacy, the original data used for the analysis are not publicly available but from the corresponding author at a reasonable request.
